# Adherence to the Mediterranean diet assessed by a novel dietary biomarker score and mortality in older adults: the InCHIANTI cohort study

**DOI:** 10.1186/s12916-021-02154-7

**Published:** 2021-11-24

**Authors:** Nicole Hidalgo-Liberona, Tomás Meroño, Raul Zamora-Ros, Montserrat Rabassa, Richard Semba, Toshiko Tanaka, Stefania Bandinelli, Luigi Ferrucci, Cristina Andres-Lacueva, Antonio Cherubini

**Affiliations:** 1 Biomarkers and Nutrimetabolomics Laboratory, Department of Nutrition, Food Sciences and Gastronomy, Faculty of Pharmacy and Food Sciences, University of Barcelona, Barcelona, Spain; 2 Centro de Investigación Biomédica en Red de Fragilidad y Envejecimiento Saludable (CIBERFES), Instituto de Salud Carlos III, Madrid, Spain; 3 Unit of Nutrition and Cancer, Cancer Epidemiology Research Programme, Catalan Institute of Oncology (ICO), Bellvitge Biomedical Research Institute (IDIBELL), Barcelona, Spain; 4 Wilmer Eye Institute, Johns Hopkins University School of Medicine, Baltimore, MD USA; 5 Translational Gerontology Branch, National Institute on Aging, NIH, Baltimore, MD 21224 USA; 6 Geriatric Unit, ASL Toscana Centro, Firenze, Italy; 7 Clinical Research Branch, National Institute on Aging, NIH, Baltimore, Maryland USA; 8 Geriatria, Accettazione Geriatrica e Centro di Ricerca per l’invecchiamento, IRCCS INRCA, Ancona, Italy

**Keywords:** Dietary biomarkers, Older adults, Mediterranean diet, Mortality, Polyphenols, Carotenoids, Dietary questionnaires

## Abstract

**Background:**

Dietary biomarkers may complement dietary intake assessment made by dietary questionnaires. We developed an a-posteriori dietary biomarkers score based on Mediterranean diet food groups and evaluated its association with mortality.

**Methods:**

642 participants (56% female), aged ≥65 years, with complete data on dietary biomarkers were followed during 20 years in the InCHIANTI cohort study (Tuscany, Italy). The main outcomes were all-cause, cardiovascular, and cancer mortality. Dietary biomarkers were selected from literature and from correlation analyses with dietary intakes of Mediterranean diet food groups in the study. The baseline levels of the following dietary biomarkers were chosen: urinary total polyphenols and resveratrol metabolites, and plasma carotenoids, selenium, vitamin B12, linolenic, eicosapentaenoic and docosahexaenoic acids, and the mono-unsaturated/saturated fatty acid ratio. Associations of the Mediterranean diet score using dietary biomarkers and a validated food frequency questionnaire (FFQ) (as tertiles) with mortality were assessed through Cox regression.

**Results:**

During the 20-year follow-up [median (Q1–Q3), 14 (8–18) years], and 435 deaths occurred (139 from cardiovascular diseases and 89 from cancer-related causes). In the fully adjusted models, the dietary biomarker-Mediterranean diet score was inversely associated with all-cause (HR_T3vs.T1_ 0.72; 95%CI 0.56–0.91) and cardiovascular (HR_T3vs.T1_ 0.60; 95%CI 0.38–0.93), but not with cancer mortality. Associations between the FFQ-Mediterranean diet score and mortality were not statistically significant.

**Conclusions:**

A greater adherence at baseline to a Mediterranean diet assessed by a dietary biomarker score was associated with a lower risk of mortality in older adults during a 20-year follow-up. The measurement of dietary biomarkers may contribute to guide individualized dietary counseling to older people.

**Trial registration:**

NCT01331512

**Supplementary Information:**

The online version contains supplementary material available at 10.1186/s12916-021-02154-7.

## Background

In 2018, there were 101.1 million persons aged >65 years (19.7%) living in Europe. In 2050, estimations predict an increase up to 149.2 million of older adults, which will represent almost 30% of the overall population [1]. Strategies to promote healthy aging are one of the pillars to minimize the health care and socio-economic impact of the increasing proportion of older adults in Europe [2]. Healthy aging can help to reduce the burden of chronic diseases, disability, and increasing health expenditure related to a longer life expectancy of older adults [3, 4].

A healthy diet is considered one of the fundamental factors to achieve healthy aging [5]. Indeed, a growing body of epidemiological evidence shows that the Mediterranean diet (MD) may delay or prevent frailty, cognitive decline, and the onset of many chronic diseases in older subjects [6–9]. Furthermore, several observational studies, including the European Prospective study into Cancer and Nutrition (EPIC)-elderly study, a cohort of 74,607 men and women aged ≥60 years, have shown inverse associations between a greater adherence to different MD scores (MDS), in both the Mediterranean and non-Mediterranean countries, and total mortality [8].

Diverse modifications or adaptations of the original MDS, initially developed by Tricophoulou et al. [10], have been applied to evaluate relationships between MD and health outcomes [11]. However, to date, adherence to MD has been almost exclusively assessed using dietary questionnaires, such as 24-h recalls and food frequency questionnaires (FFQ), which are susceptible to random and systematic errors in estimating dietary intake [12]. In addition, age-related changes in the digestion and absorption of foods and nutrients could introduce further bias into the accurate assessment of the relationships between dietary intakes and health outcomes in older adults. In our previous analyses from the *Invecchiare nel Chianti* (InCHIANTI) study, no association was observed between either dietary total polyphenol or polyunsaturated fatty acid (PUFA) intakes and all-cause mortality. However, statistically significant inverse associations were found with their dietary biomarkers: total urinary polyphenols [13] and serum PUFA concentrations [14], respectively. Both dietary biomarkers are directly related to key features of a MD pattern. Total urinary polyphenol concentrations positively correlate with plant-based foods, such as vegetables, fruits, and nuts [15], while plasma PUFA levels positively correlate with fish and seafood consumption [16]. Thus, the use of dietary biomarkers may improve the estimations of MD exposure during a long-term follow-up [17]. Other relevant candidates to be included as a dietary biomarker in a panel correlated with MD are plasma levels of carotenoids and selenium [18, 19]. In particular, total carotenoids have been shown as a relevant dietary biomarker for the consumption of vegetables, fruits, cereals, and nuts significantly associated with their health-promoting effects [19]. Recently, Li et al. captured a metabolomics signature related with dietary MDS (based on 67 endogenous metabolites) that was inversely associated with incident cases of cardiovascular disease (CVD) in a Spanish and 3 US cohorts, even after adjustment for the dietary MDS from it was developed [20]. These findings give further support to the hypothesis that biomarkers are better correlated with the overall health-promoting effects of MD.

The current research aims at developing a dietary biomarker panel based on key MD food groups in the population from the InCHIANTI study and investigating its long-term association with all-cause, CVD, and cancer mortality. We also compared mortality prediction using dietary biomarker-MDS and FFQ-MDS.

## Methods

### Study design

The InCHIANTI study is an ongoing prospective cohort of a representative sample of older adults living in the Chianti geographic area (Tuscany, Italy). It was designed to evaluate factors that influence mobility and disability in late adulthood [21]. Details of the InCHIANTI study have been previously published [21]. Participants were recruited in 1998–2000 and were invited every 3 years to a follow-up visit. The Italian National Institute of Research and Care of Aging Ethical Committee approved the study protocol, and all participants signed an informed participation consent.

The current study was conducted and reported in accordance with the Strengthening the Reporting of Observational Studies in Epidemiology-Nutritional Epidemiology (STROBE-NUT) guidelines (Additional file 1. Supplementary Table S1, [22]).

### Study population

At baseline, 1155 subjects aged ≥65 years agreed to participate, with a participation rate of 91.7%. Out of these, participants who had missing data in the FFQ (*n*=16), in any of the selected dietary biomarkers (*n*=472) or covariates of interest (*n*=21), were excluded. The major cause of missing data in dietary biomarkers was the failure to complete the baseline 24h urine collection.

### Dietary assessment

Habitual dietary intake was assessed at baseline by trained interviewers using the Italian version of the FFQ developed and validated in the EPIC-Italy study [23]. This questionnaire asked how often (daily, week, monthly) the consumption of 198 food and beverages items in the past year, considering its respective portions sizes. Daily intake of energy, macronutrients, and micronutrients was estimated from the dietary questionnaire using a specific software developed for the EPIC study [24]. For the current analysis, dietary data were available at baseline, 3, 6, and 9 years of follow-up.

#### Dietary score of the Mediterranean diet

Adherence to a dietary MDS was computed using an 18-point linear scale that incorporated 9 key components of the diet. Each component was divided into tertiles of intakes, and a score of 0, 1, and 2 was assigned to the first, second, and third tertiles of intake for the 6 components presumed to fit the MD: vegetables, legumes, fruits and nuts, cereals, fish, and ratio monounsaturated fatty acids (MUFA)/saturated fatty acids (SFA). Alcohol was scored as a dichotomous variable, assigning 2 for moderate consumers (range 5–25 g/days for women and 10–50 g/days for men) and 0 for subjects above or below the sex-specific range, including teetotallers. The scoring was inverted for the 2 components presumed to not fit the MD: total meat and dairy products. The overall adherence to MDS from dietary intakes (FFQ-MDS) was calculated for each subject as the sum of the values from each component, which resulted in a score between 0 (lowest adherence) and 18 (highest adherence) [25].

### Nutritional biomarker assessment

For this study, the measurement of dietary biomarkers was only available at baseline. Plasma carotenoids were measured using high-performance liquid chromatography (HPLC). Total carotenoids were calculated as the sum of α-carotene, β-carotene, β-cryptoxanthin, lutein, zeaxanthin, and lycopene in micromoles per liter (μmol/L). Within-run and between-run coefficients of variation, respectively, were 7.3% and 9.6% for α-carotene, 4.5% and 5.4% for β-carotene, 2.7% and 3.5% for β-cryptoxanthin, 2.6% and 7.1% for lutein, 6.2% and 6.8% for zeaxanthin, and 7.5% and 7.8% for lycopene [26, 27].

Selenium concentration (μmol/L) at baseline was measured by graphite furnace atomic absorption spectrometry with an Analyst 600 with Zeeman background correction (Perkin Elmer, Norwalk, CT). For baseline measurements, the instrument was calibrated daily by using known plasma selenium standards (UTAK Laboratories Inc., Valencia, CA). Within-run and between-run CVs were 3.1% and 7.1%, respectively [28].

Plasma fatty acids (FAs) were measured by gas chromatography (HP-6890, Hewlett-Packard, Palo Alto, CA, USA) with a fused silica capillary column (30 m × 0.25 mm internal diameter, HP-225 from Hewlett-Packard, Palo Alto, CA, USA). Total lipids were extracted from 0.15 mL of the plasma by using the procedure of Folch (1957). A known amount (50 μg) of heptadecanoic acid (C17:0, Sigma Chemical Co., St. Louis, MO, USA) was added to each sample before extraction as an internal standard. Fatty acid methyl esters (FAMEs) were prepared through transesterification using Lepage and Roy’s method, modified according to Rodriguez-Palmero et al. (1998). FAMEs were identified by comparison with pure standards (Nu-Chek Prep, Inc., Elysian, MN, USA), and peaks were identified by comparison with standard mixtures of fatty acids. For quantitative and qualitative analysis of fatty acids as methyl esters, calibration curves for FAME (ranging from C14:0 to C24:1) were prepared by adding six increasing amounts of individual FAME standards to the same amount of internal standard (C17:0; 50 μg). The correlation coefficients for the calibration curves of 20 fatty acids were in all cases higher than 0.998 in the range of concentrations studied. The amount of plasma fatty acids (ranging from C14:0 to C24:1) was quantified based on the amount of FAME internal standard (C17:0) that was recovered. The coefficient of variation for all fatty acids was on average 1.6% for intraassay and 3.3% for interassa y[29].. The percentage of values below the limit of detection were 33% for C24:0 (tetracosanoic acid), 14% for C20:0 (eicosanoic acid), 5% for C22:1 n-9 cis (docosenoic acid), and 3% for C22:0 (docosanoic acid). In these cases, samples were assigned with the minimum detectable value (0.15 μM)

Serum vitamin B12 was measured at baseline using by radioligand-binding assay (SimulTrac-SNB Radio- assay; ICN Pharmaceuticals). The minimum detectable concentrations were 75 ng/L for vitamin B12, and the intraassay and interassay CVs were 11% and 12%, respectively [30].

In 24h urine samples, total polyphenol concentration was measured by the Folin-Ciocalteau assay after a solid-phase clean-up which allows the elimination of interfering substances that could react with the F-C assay, as described previously [31]. Total polyphenol concentrations were expressed as milligrams of gallic acid equivalents (GAE) per 24-h urine. Phase II resveratrol metabolites were measured by a liquid chromatography-tandem mass spectrometry (LC-MS/MS) as previously described [32]. Briefly, 1mL of urine with the internal standard was loaded into a previously equilibrated Oasis (Waters) HLB (hydrophilic-lipophilic-balanced) solid-phase extraction 96-well plate (30 mg). Urinary resveratrol metabolites were eluted with acidified methanol solution and ethylacetate. After evaporation, samples were reconstituted with 100 μL of the mobile phase and then analyzed by liquid chromatography (PerkinElmer S200) coupled to a triple-quadrupole mass spectrometer (API3000; AppliedBio-systems). Intra-batch and inter-batch coefficients of variation were less than 10.5% and less than 10.7%, respectively [32]. Both plasma and urinary dietary biomarkers were already validated against dietary intake measurements in the InCHIANTI study [28–31, 33, 34]. In the case of urinary resveratrol, 31% of the samples had values below the limit of detection. These belonged mostly to teetotallers (56%) and participants who did not consume wine (26%). A zero value was assigned to all these samples.

#### Biomarker score of the Mediterranean diet

The following dietary biomarkers were considered: total carotenoids (calculated as the sum of α-carotene, β-carotene, β-cryptoxanthin, lutein, zeaxanthin, and lycopene), selenium, linoleic acid, eicosapentaenoic (EPA) and docosahexaenoic acids (DHA), MUFAs [calculated by summing of the following fatty acids: C14:1 n-9 cis (myristoleic acid), C16:1 n-7 cis (palmitoleic acid), C18:1 n-9 cis (oleic acid), C18:1 n-7 trans (octadecenoic acid), C20:1 n-9 cis (11-eicosenoic), C22:1 n-9 cis (docosenoic acid), and C24:1 n-9 cis (tetracosenoic acid)], SFAs [calculated as the sum of C14:0 (myristic acid), C16:0 (palmitic acid), C18:0 (stearic acid), C20:0 (eicosanoic acid), C22:0 (docosanoic acid), and C24:0 (tetracosanoic acid)], and vitamin B12.

Similar to the FFQ-MDS, the dietary biomarker-MDS was computed using an 18-point linear scale that incorporated a dietary biomarker from 9 key components of the diet. From the available measurements in the InCHIANTI database, we selected those that were suggested in previous literature as a dietary biomarker of the key MD food groups [16, 18, 35–40] and in addition were significantly associated with dietary intake data in the present study (shown in Table 1). Dietary biomarkers for vegetables, legumes, fruits and nuts, cereals, fish, and olive oil were ranked and divided by tertiles. A score of 0, 1, and 2 was assigned to the first, second, and third tertiles of dietary biomarker, respectively. Resveratrol metabolites as a dietary biomarker of alcohol consumption were scored as a dichotomous variable, assigning 2 for moderate consumers (range of values corresponding to wine consumption; 125–375 g/day for men and 50–250 g/days for women; in the present population 589–14,557 nmol/24h for men and 1–11,125 nmol/24h for women) [39] and 0 for subjects above or below the sex-specific range, including teetotallers. The wine was the major contributor to alcohol intake (88%) in this older Mediterranean population. The scoring was inverted for the SFA and vitamin B12 tertiles, representing meat and dairy products, respectively. Dietary biomarker-MDS ranged from 0 to 18, indicating low to high adherence.

**Table 1:** Mediterranean diet adherence score (MDS) by dietary components and biomarkers

Score	MDS		Spearman’s rank correlation coefficient
	Dietary components (FFQ)	Dietary biomarkers (dBMK)	
Tertiles (0,1,2)	Vegetables Legumes Fruits and nuts Cereals	Total polyphenols Carotenoids Linolenic acid Selenium	0.170 (*P*<0.001)
Tertiles (0,1,2)	Fish	EPA+DHA	0.177 (*P*<0.001)
Tertiles (0,1,2)	MUFA/SFA	MUFA/SFA	0.229 (*P*<0.001)
Tertiles (0,2,0)	Alcohol	Resveratrol	0.668 (*P*<0.001)
Tertiles (2,1,0)	Meat	SFA	0.109 (*P*=0.005)
Tertiles (2,1,0)	Dairy products	Vitamin B12	0.135 (*P*=0.001)
Total score (0–18)	All dietary components	All biomarkers	0.263 (*P*<0.001)

*MDS* Mediterranean diet adherence score, *FFQ* food-frequency questionnaire, *EPA* eicosapentaenoic acid, *DHA* docosahexaenoic acid, *MUFA* monounsaturated fatty acids, *SFA* saturated fatty acid

### Genetic factors related with mortality and parental longevity score

Overnight fasted blood samples were used for genomic DNA extraction as previously described [41]. Illumina Infinium HumanHap 550K SNP arrays were used for genotyping of the following single nucleotide polymorphism (SNP)s: *APOE* ε4 (using the rs429358 and rs7412 SNPs), rs1421783 *MAT2B*, rs6997892 *WRN*, rs10817931 *TRIM32*, rs2684766 *IGF1R*, and rs11630259 *IGF1R* [42]. The parental longevity score was created from the parental age at death or current age (if alive) as described previously [43]. Briefly, a normal curve using a non-linear least square regression was used to determine the modal age (M) of death for each parent. They were then categorized as short-lived if *M* was less than M− 1 standard deviation (mothers 61–76 years and fathers 46–74 years), intermediate as M± 1 standard deviation (mothers 77–91 years and fathers 75–87 years), and long-lived (mothers: older than 91 years and fathers: older than 87 years).

### Outcome assessment

Data on 20-year mortality were collected using the Mortality General Registry maintained by the Tuscany Region, as well as death certificates delivered after participants’ decease to the registry office of the municipality of residence [13]. Cardiovascular mortality, based on the underlying cause of death, was defined as any cardiovascular mortality coded by the 9th Revision of the International Classification of Diseases (ICD-9, codes 390-459). Cancer mortality was defined as any mortality related to known cancer (coded 140 to 239 by the ICD-9). Other mortality causes (also coded by ICD-9) included respiratory system diseases; unknown causes; injury and poisoning; nervous system and sense organ diseases; endocrine, nutritional and metabolic diseases, and immunity disorders; mental disorders; digestive system diseases; symptoms, signs, and ill-defined conditions; infectious and parasitic diseases; blood and blood-forming organ diseases; and musculoskeletal system and connective tissue diseases. Cases lost during follow-up (i.e., emigration or refusal to participate) were censored using the date of the last contact.

### Other main baseline covariates assessment

Covariates were selected a priori on the basis of previously reported associations with both MD and mortality [12, 15, 37]. Trained interviewers administered standardized questionnaires on sociodemographic and lifestyle variables including age, sex, and years of education. Smoking habits were self-reported, and participants were classified into never smokers, former smokers, and current smokers. Physical activity was evaluated using a structured questionnaire specifically developed and validated for the InCHIANTI study. The questionnaire required that the participant provide data on past and current physical activity. The details of the questionnaire have been previously reported [44]. Physical activity was coded into the following categories: inactive or sedentary (physical activity <2 h/week; i.e., walking), light physical activity (2–4 h/week), and moderate-high physical activity (light-intensity activity >4 h/week or moderate-intensity activity 1–2 h/week; i.e., swimming) [44]. Height and weight were measured, and body mass index (BMI) was computed into kg/m^2^. Comorbidities included in this analysis were diabetes mellitus (type 1 or type 2), hypertension (HT), chronic obstructive pulmonary disease (COPD), cardiovascular disease (CVD, including angina, myocardial infarction, congestive heart failure, and stroke), impaired renal function (glomerular filtration rate <60 ml/min), Parkinson’s disease, dementia, and cancer. They were defined using standard clinical definitions by combining information from self-reported physician diagnoses, pharmacological treatments, medical history, clinical examinations, and blood tests [45].

### Statistical analysis

Descriptive analysis of baseline characteristics was presented as mean (standard deviation) for normally distributed variables or median (25th and 75th percentiles) for variables that deviated from the normal distribution. Spearman rank correlation analyses were performed to examine the relations between proposed dietary biomarkers and MD diet food groups and between FFQ-MDS and dietary biomarker-MDS. The final sum of both scores was divided into population tertiles to achieve categories with a similar number of participants in each group. Cut-offs for FFQ-MDS tertiles were ≤7, 8–10, and ≥11 and for dietary biomarker-MDS tertiles ≤8, 9–10, and ≥11. The squared-weighted Kappa coefficient was calculated as a measure of the agreement between FFQ-MDS and dietary biomarker-MDS tertiles. Baseline characteristic comparisons across the FFQ-MDS and dietary biomarker-MDS tertiles were assessed using generalized linear models adjusted for age and sex.

Cox proportional hazard models were used to evaluate the associations between tertiles of FFQ-MDS or baseline dietary biomarker-MDS and all-cause, cardiovascular, and cancer mortality. The base model was adjusted for age (continuous) and sex. The final model was additionally adjusted for BMI (continuous); years of education (continuous); smoking status (3 categories); physical activity (3 categories); impaired renal function, diabetes mellitus, HT, COPD, CVD, cancer, dementia, and Parkinson’s disease (dichotomous); and energy intake (continuous). Similarly, each component of the dietary biomarker-MDS (as tertiles) was individually tested in the fully adjusted model. Tests for linear trends were performed by assigning ordinal scores to the tertiles. For linear dose-response plots, Cox regression models were carried out with dietary biomarker MDS or FFQ-MDS as continuous variables using the “rms” R package developed by Frank Harrell [46].

Interactions between FFQ-MDS and dietary biomarker-MDS (as tertiles) and age (< or ≥80years), sex, BMI categories (< 25 kg/m^2^, 25–30 kg/m^2^, and >30 kg/m^2^), smoking status (never, former, and current smokers), HT, CVD, impaired renal function, diabetes mellitus, COPD, and cancer in relation to total, cardiovascular, and cancer mortality were evaluated in the fully adjusted model using the likelihood ratio test. Sensitivity analyses were run after the exclusion of participants who died in the first 2 years of the follow-up, or participants using dietary supplements or lipid-lowering medications. In all Cox models, proportional hazard assumption was tested by visual inspection of the plots based on the Schoenfeld residuals and they were satisfied.

In addition, to better understand genetically predisposed mortality risks, we further adjusted the Cox regression models for SNPs with previously reported associations with mortality [42]: *APOE* ε4 (using the rs429358 and rs7412 SNPs), rs1421783 *MAT2B*, rs6997892 *WRN*, rs10817931 *TRIM32*, rs2684766 *IGF1R*, rs11630259 *IGF1R*, and a parental longevity score.

Linear mixed models were used to check for differences in the FFQ-MDS during the repeated measures of the study using individual-specific random effects. Fixed categorical factors were interview number (4 levels: baseline, 3, 6, and 9 years of follow-up) and sex, and continuous covariates were age and energy intake. Mixed effect Cox regression models with time-dependent covariates were used to test the FFQ-MDS relationship with all-cause, cardiovascular, and cancer mortality including the dietary data collected at baseline, 3, 6, and 9 years of follow-up in the base and the fully adjusted models.

SPSS statistical software 25.0 (IBM, USA) and R version 3.2.3 (R Foundation for Statistical Computing, Austria) were used for all statistical analyses. *P* values (two-tailed) <0.05 were considered statistically significant.

## Results

### Descriptive analysis

**Fig. 1:**
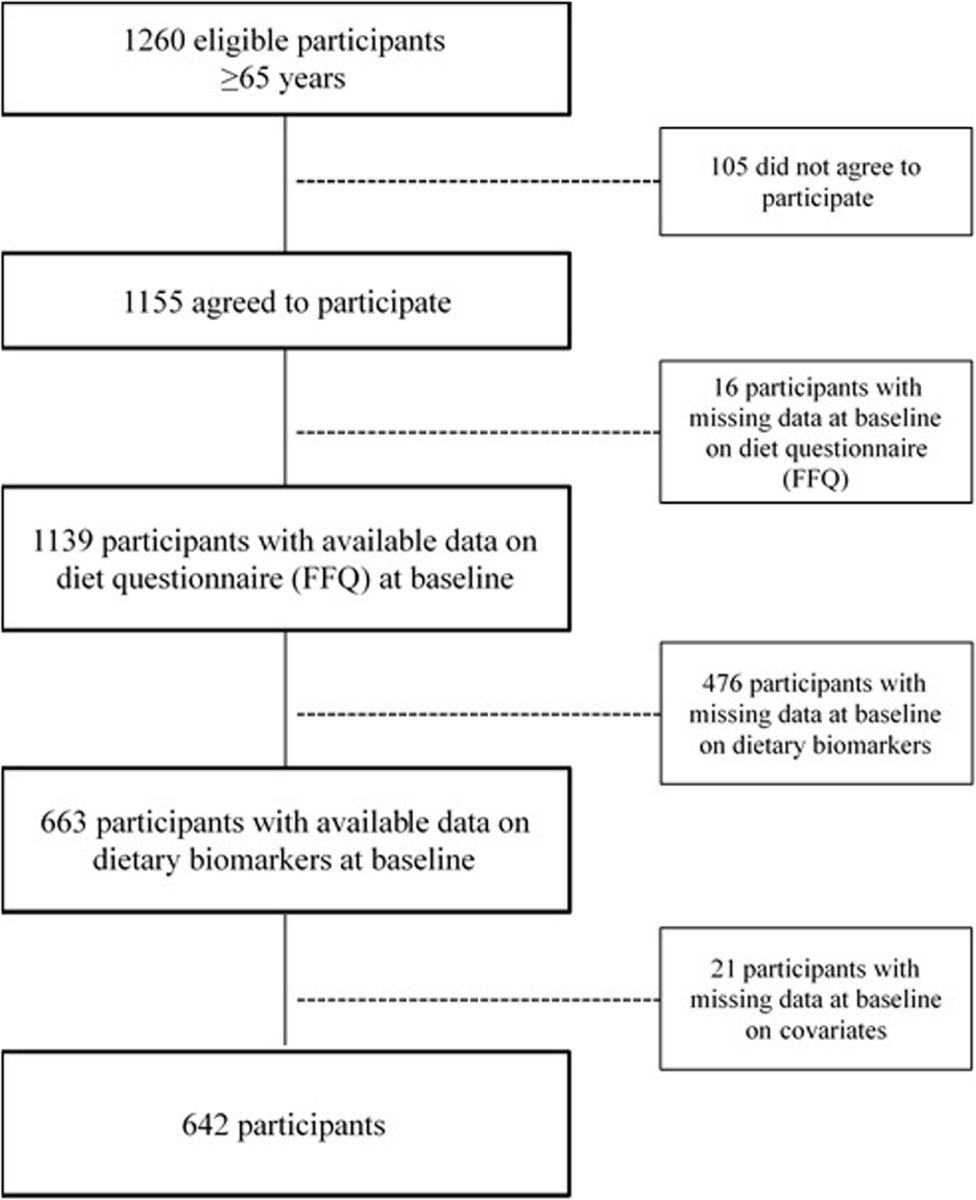
Flowchart of participants of the study

Out of the 1155 participants surveyed at baseline, 642 [357 women and 285 men, with a mean (SD) age of 74±7 years], were included in the study (Fig. 1). The main cause of exclusion from the study was not collecting 24h urine specimens (472 out of 513). These 513 participants were slightly older (77 vs. 74 years), with fewer years of education (5.1 vs*.* 5.4) and showed a higher prevalence of low physical activity (33% vs. 17%), dementia (11.3% vs. 3.7%), and Parkinson’s disease (2.2% vs. 0.8%), as well as lower prevalence of HT (48% vs. 63%) than the 642 participants included in this study (all *p*<0.05). Among the 642 selected participants, HT and impaired renal function were the most common comorbidities at baseline with a prevalence of 63% and 39%, respectively (Table 2), followed by CVD (23%) and diabetes mellitus (14%).

**Table 2:** Baseline characteristics of the study population by dietary biomarker-MDS and FFQ-MDS tertiles

Characteristics	All (***n***=642)	Dietary biomarker-MDS			P*	FFQ-MDS			P*
		Tertile 1 (***n***=251)	Tertile 2 (***n***=193)	Tertile 3 (***n***=198)		Tertile 1 (***n***=191)	Tertile 2 (***n***=265)	Tertile 3 (***n***=186)	
Age at baseline (years) ^a^	74 (7)	75 (7)	75 (7)	73 (6)	0.013	76 (7)	75 (7)	72 (5)	<0.001
Female sex (*n*,%)	357 (56)	145 (58)	105 (54)	107 (54)	0.82	130 (68)	155 (58)	72 (39)	<0.001
BMI (kg/m^2^) ^a^	27.4 (3.9)	27.4 (4.3)	27.9 (3.7)	27.0 (3.4)	0.19	27.4 (4.3)	27.4 (3.7)	27.6 (3.6)	0.67
Education (years) ^a^	5.4 (3.3)	5.1 (2.6)	5.6 (3.6)	5.7 (3.6)	0.19	5.1 (3.1)	5.3 (3.2)	6.0 (3.4)	0.58
Smoking (*n*,%)					0.001				0.12
Never	382 (60)	1147 (59)	115 (60)	120 (61)		122 (64)	167 (63)	93 (50)	
Former	174 (27)	58 (23)	54 (28)	62 (31)		44 (23)	65 (25)	65 (35)	
Current	86 (13)	46 (18)	24 (12)	16 (8)		25 (13)	33 (12)	28 (15)	
Physical activity (*n*, %)					0.034				0.044
Sedentary	110 (17)	52 (21)	437 (19)	21 (11)		49 (26)	44 (17)	17 (9)	
Light	281 (44)	115 (46)	78 (40)	88 (44)		87 (46)	117 (44)	77 (41)	
Moderate-High	251 (39)	84 (33)	78 (40)	89 (45)		55 (29)	104 (39)	92 (49)	
Energy intake (kcal/day) ^a^	1928 (543)	1833 (511)	1979 (546)	1998 (564)	0.003	1751 (539)	1905 (515)	2141 (515)	<0.001
HT (*n*,%)	402 (63)	157 (62)	124 (64)	121 (61)	0.96	131 (66)	165 (62)	106 (56)	0.19
IRF (*n*,%)	253 (39)	104 (41)	71 (37)	78 (39)	0.31	92 (48)	116 (44)	45 (24)	0.16
DM (*n*,%)	89 (14)	44 (18)	24 (12)	21 (11)	0.023	29 (15)	39 (15)	21 (11)	0.38
COPD (*n*,%)	49 (8)	24 (10)	14 (7)	11 (6)	0.05	15 (8)	18 (7)	16 (9)	0.25
CVD (*n*,%)	147 (23)	66 (26)	45 (23)	36 (18)	0.06	44 (23)	63 (24)	40 (22)	0.76
Cancer (*n*,%)	39 (6)	19 (8)	9 (5)	11 (6)	0.44	13 (7)	18 (7)	8 (4)	0.55
Dementia (*n*,%)	24 (4)	12 (5)	8 (4)	4 (2)	0.22	10 (5)	8 (3)	6 (3)	0.52
Parkinson’s disease (*n*, %)	5 (0.8)	1 (0.4)	1 (0.5)	3 (1.5)	0.24	-	3 (1.1)	2 (1.0)	0.93

*BMI* body mass index, *IRF* impaired renal function, *DM* diabetes mellitus, *COPD* chronic obstructive pulmonary disease, *HT* hypertension, *CVD* cardiovascular disease. Cut-offs for FFQ-MDS tertiles were ≤7, 8–10, and ≥11; and for dietary biomarker-MDS tertiles: ≤8, 9–10, and ≥11. These cutoffs were chosen to achieve 3 categories with a similar number of participants in each group

*p values calculated using generalized linear models adjusted for age and sex

^a^Data reported as mean (SD)

The correlations among dietary components of FFQ-MDs and concentrations of a dietary biomarker in the population are presented in Table 1. For the dietary biomarker-MDS, we grouped the categories of vegetables, fruits and nuts, legumes, and cereals because the selected dietary biomarker (i.e., total polyphenols and carotenoids) were ubiquitously distributed among these food groups. Alcohol intake in the FFQ-MDS and urine resveratrol in the dietary biomarker-MDS were highly correlated. The total FFQ-MDS (0-18) was moderately correlated with the dietary biomarker-MDS (*r*=0.26), and the level of agreement between the classifications of FFQ-MDS and dietary biomarker-MDS tertiles was relatively low [squared-weighted Kappa coefficient (95% CI) = 0.218 (0.164–0.272)].

The characteristics of the study population categorized by dietary biomarker-MDS and FFQ-MDS tertiles are shown in Table 2. Participants in the highest tertile of both dietary biomarker-MDS and FFQ-MDS were younger and more likely to have higher energy intake and being more physically active than those in the lowest tertile. In addition, participants in the highest dietary biomarker-MDS tertile showed a lower proportion of current smokers and diabetes mellitus at baseline, while subjects in the highest FFQ-MDS tertile were predominantly men compared to those at the lowest tertile. Dietary intakes of food groups and concentrations of dietary biomarkers according to dietary biomarker-MDS and FFQ-MDS tertiles are shown in Additional file 1, Supplementary Tables S2 and S3, respectively.

### Association between Mediterranean diet exposure and mortality

**Fig. 2:**
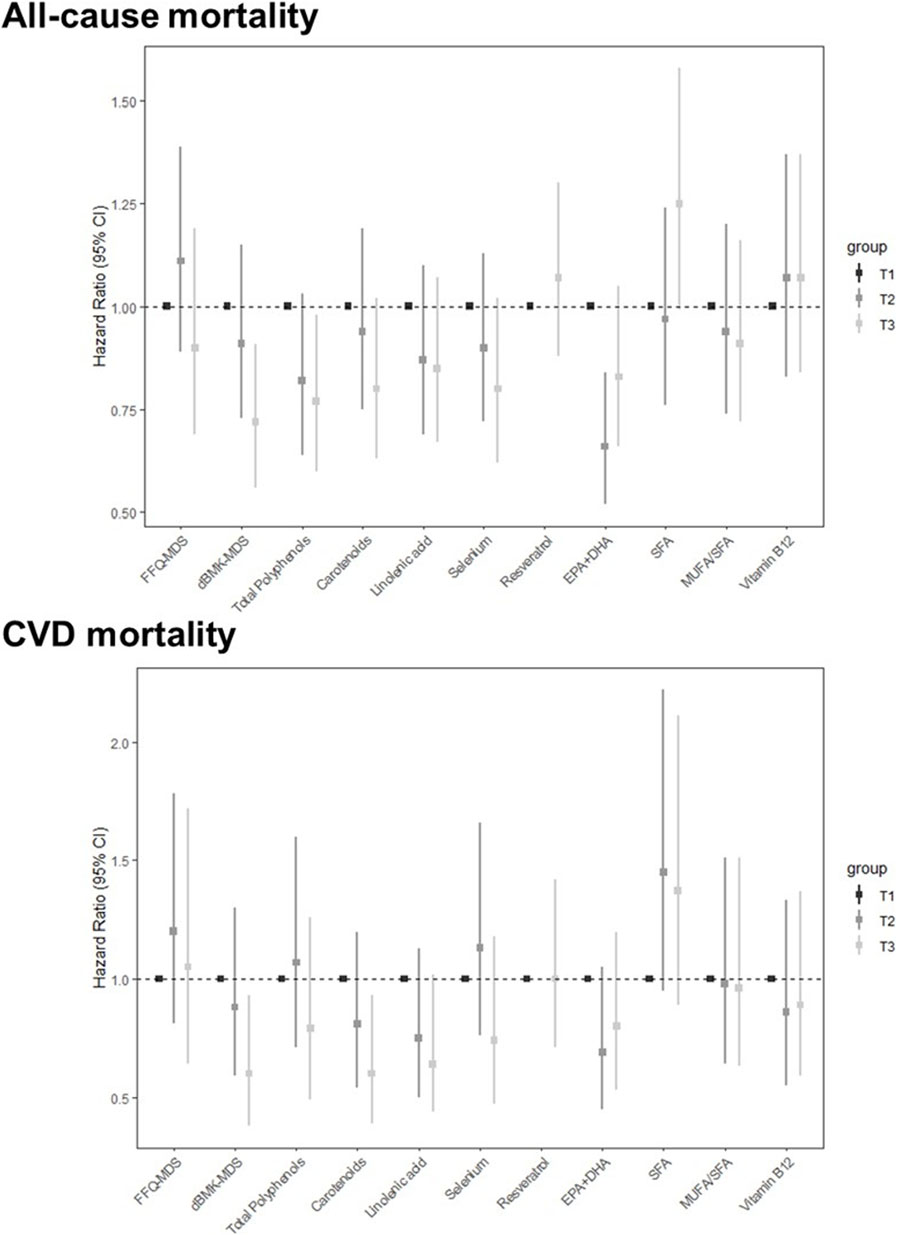
Association between FFQ- and dietary biomarker-MDS and individual dietary biomarkers (as tertiles), and all-cause, CVD, and cancer mortality in the InCHIANTI Study. Cox regression model included sex, age, BMI, education, smoking status, physical activity, impaired renal function, diabetes mellitus, chronic obstructive pulmonary disease, hypertension, cardiovascular disease, cancer, dementia, Parkinson’s disease, and energy intake. FFQ food frequency questionnaire, dBMK dietary biomarker, EPA eicosapentaenoic acid, DHA docosahexaenoic acid, MUFA monounsaturated fatty acids, SFA saturated fatty acids. The total number of deaths, 435; CVD deaths, 139; cancer deaths, 85. Resveratrol was categorized into two groups: moderate vs*.* no or high consumers.

During the 20 years of follow-up (median 14 years, Q1–Q3: 8–18 years), 435 deaths occurred (139 attributed to CVD and 85 to cancer-related causes). In the base models, a greater adherence to dietary biomarker-MDS at baseline was significantly associated with a lower all-cause mortality (HR_T3vs.T1_ 0.66; 95%CI 0.52, 0.83), and this association remained statistically significant in the fully adjusted model (HR_T3vs.T1_ 0.72; 95%CI 0.56, 0.91) (Fig. 2, and Additional file 1, Supplementary Table S4). Moreover, the dietary biomarker-MDS showed a linear dose-response relationship with overall mortality [(HR per unit increase 0.96; 95%CI 0.83, 0.99); Additional file 1, Supplementary Table S4 and Supplementary Fig S1]. The FFQ-MDS was inversely, but not significantly, associated with all-cause mortality either in the base model (HR_T3vs.T1_ 0.91; 95%CI 0.70, 1.19) or in the fully adjusted model (HR_T3vs.T1_ 0.90; 95%CI 0.69, 1.19) (Fig. 2, and Additional file 1, Supplementary Table S4). Similarly, no linear association was observed between FFQ-MDS and overall mortality [(HR per unit increase 1.01; 95%CI 0.97, 1.05); Additional file 1, Supplementary Table S4 and Supplementary Fig S1].

Each component of the dietary biomarker-MDS at baseline was individually tested for its relationship with overall mortality (Fig. 2, and Additional file 1, Supplementary Table S1). Baseline urinary total polyphenols were significantly and inversely associated with all-cause mortality in the fully adjusted model (HR_T3vs.T1_ 0.77; 95%CI 0.60, 0.98, *p*=0.036). Plasma concentrations of carotenoids (*p*=0.076), selenium (*p*=0.068), and plasma SFA levels (*p*=0.059) were negatively associated with overall mortality, but without achieving statistical significance. Moreover, a linear inverse association with all-cause mortality was observed for linolenic acid (HR per log-unit increase 0.62; 95%CI 0.40, 0.95) and EPA+DHA (HR per log-unit increase 0.54; 95%CI 0.30, 0.99) (Supplementary Table S4).

Similar results were obtained when CVD mortality was defined as an outcome (Additional file 1, Supplementary Table S5). While the dietary biomarker-MDS was inversely associated with CVD mortality in the fully adjusted model (HR_T3vs.T1_ 0.60; 95%CI 0.38, 0.93), the FFQ-MDS was not (HR_T3vs.T1_ 1.05; 95%CI 0.64, 1.72, Fig. 2). Likewise, the dietary biomarker-MDS showed a statistically significant linear association with CVD mortality (HR per unit increase 0.93; 95%CI 0.87, 0.99), while the FFQ-MDS did not (HR per unit increase 0.99; 95%CI 0.94, 1.07) (Additional file 1, Supplementary Table S5). Among the individual components of the dietary biomarker-MDS, baseline total plasma carotenoid concentrations were significantly associated with CVD mortality (HR_T3vs.T1_ 0.60; 95%CI 0.39, 0.93), while linolenic acid showed an inverse marginal association (*p*=0.064, Fig. 2, and Additional file 1, Supplementary Table S5). Linear inverse associations with CVD mortality were observed for linolenic acid (HR per log-unit increase 0.31; 95%CI 0.15, 0.66), selenium (HR per log-unit increase 0.09; 95%CI 0.01, 0.76), and SFA (HR per log-unit increase 0.17; 95%CI 0.03, 0.96) (Additional file 1, Supplementary Table S5). No significant association was observed between either any MDS or dietary biomarker individual component and cancer mortality (all *p*>0.05, Fig. 2, and Additional file 1, Supplementary Table S6).

Interactions between age, sex, BMI, smoking status, HT, CVD, diabetes mellitus, cancer, and both the FFQ-MDS and the dietary biomarker-MDS in relation to all-cause, CVD, or cancer mortality were mostly not significant. There was a significant interaction between FFQ-MDS and COPD (*p*=0.017) by which its association with all-cause mortality was only significant in patients with COPD (*n* 53, deaths 40; HR_T3vs.T1_ FFQ-MDS 0.24; 95%CI 0.06, 0.93). On the other hand, a significant interaction was noticed between the dietary biomarker-MDS and impaired renal function for its association with all cause-mortality (*p* for interaction=0.031). The association between the dietary biomarker-MDS and all-cause mortality remained significant only among the participants without impaired renal function at baseline (*n* 390, deaths 226; HR_T3vs.T1_ dietary biomarker-MDS 0.56; 95%CI 0.39, 0.80). In those with impaired renal function, this association was not statistically significant (*n* 253, deaths 212; HR_T3vs.T1_ dietary biomarker-MDS 0.94; 95%CI 0.67, 1.33). For CVD mortality, the only statistically significant interaction detected was between dietary biomarker-MDS and BMI (*p*=0.022). The inverse association between dietary biomarker-MDS and CVD mortality was stronger among participants with BMI>30 kg/m^2^ (*n* 161, CVD deaths 36; HR_T3vs.T1_ dietary biomarker-MDS 0.28; 95%CI 0.09, 0.90).

The inverse associations between dietary biomarker-MDS and all-cause and CVD mortality were confirmed in the sensitivity analyses after exclusion of participants who died in the first 2 years of follow-up (HR_T3vs.T1_ dietary biomarker-MDS 0.71; 95%CI 0.55, 0.90; and HR_T3vs.T1_ dietary biomarker-MDS 0.59; 95%CI 0.37, 0.94; for all-cause and CVD mortality, respectively). Further sensitivity analyses after the exclusion of participants using dietary supplements (3.3%, *n*=21) and lipid-lowering medications (3.9%, *n*=25) were computed and the results remained similar.

In addition, we further adjusted the Cox regression models for a genetic score (including APOE ε4, among other SNPs) and a parental longevity score to account for genetically predisposed mortality risk. In these models, the association between dietary biomarker-MDS and all-cause mortality was HR_T3vs.T1_ 0.70; 95%CI 0.54, 0.90, and between the dietary biomarker-MDS and CVD mortality was HR_T3vs.T1_ 0.57; 95%CI 0.35, 0.91.

The intraclass correlation coefficient (ICC) of the FFQ-MDS between follow-ups (0, 3, 6, and 9 years) was 0.49 (95% CI 0.44, 0.52). A statistically significant difference of the FFQ-MDS between the baseline and the 9-year examination (*β* 0.26; 95%CI 0.20, 0.50) was observed, but not among the other follow-up times. After including data from all follow-up dietary assessments in the analysis, we observed a significant association between the FFQ-MDS and all-cause mortality in the base model [HR_T3vs.T1_ 0.77; 95%CI 0.60, 0.99), but not in the fully adjusted model (HR_T3vs.T1_ 0.81; 95%CI 0.63, 1.04). The FFQ-MDS, including the repeated measures, was inversely associated with CVD mortality in both the base (HR_T3vs.T1_ 0.59; 95%CI 0.37, 0.93) and the fully adjusted models (HR_T3vs.T1_ 0.62; 95%CI 0.39, 0.99). For cancer mortality, no significant associations were observed with any model.

## Discussion

In the present study, a baseline dietary biomarker score based on key MD food groups but not a MDS based on the FFQ was inversely associated with long-term all-cause and CVD mortality in a cohort of older adults (median follow-up 14 years). These findings strongly suggest that a panel of dietary biomarkers may provide a more objective and accurate assessment of the health benefits associated with diet quality in older adults than self-reported questionnaires. This dietary biomarker panel can be used in both epidemiological and clinical research to further investigate the relationships between the adherence to MD and health outcomes.

Our results showing a non-significant association between FFQ-MDS and all-cause mortality somewhat contrast with previous findings from the EPIC [8], MOLI-SANI [7], and healthy aging: a longitudinal study in Europe (HALE) [47] and Women’s Health Initiative (WHI) [48] studies. These differences could be due to the older mean age of our population, the lower number of participants included, the longer follow-up [14years vs. 8.1years (in the EPIC and MOLI-SANI studies)], the higher proportion of deaths [68% vs. 10–17% (in the EPIC and MOLI-SANI)], differences on dietary backgrounds when comparing studies from the Mediterranean vs*.* non-Mediterranean regions, or on the relatively higher presence of chronic conditions like CVD at baseline, among other factors. Older age might affect the ability to report food intake using FFQ, which depends on memory, and this could hamper the accurate estimation of the associations between dietary intakes and health outcomes [49]. Moreover, dietary intakes can change over time, and therefore, the association between FFQ-MDS, measured at baseline, and long-term mortality could be inaccurate. However, the intraclass correlation coefficient of FFQ-MDS was acceptable across the consecutive examinations (0.49). Moreover, although the participants were older over the consecutive interviews, we observed minor differences in the adherence to FFQ-MDS, which was only statistically significant when comparing the first and the last evaluation. The consideration of data from the dietary assessments of the follow-ups showed similar results for overall mortality; but for CVD mortality, including the dietary data from the follow-ups did show a statistically significant inverse association between FFQ-MDS and CVD mortality.

Recently, two metabolomics studies discovered a plasma metabolite panel based on the MD adherence including more than 60 metabolites of which > 60% were lipids (such as phospholipids, glycerolipids, carnitines, and acylcarnitines) [20, 50]. Both studies used an a posteriori approach to explore metabolite fingerprints, which were significantly correlated with the MDS adherence from dietary questionnaires. In the present study, we included metabolites derived from the dietary sources, i.e., total polyphenols, resveratrol, or carotenoids, which were not considered in the abovementioned metabolomics analyses. In the study of Li et al. [20]*,* fruits and legumes were only slightly correlated with 7 out of the 67 metabolites that constituted the total score. Therefore, these metabolites may track the biological changes induced by a MD (biomarkers of effect) but may not correlate with the intake of certain major food groups of the MD. Indeed, in both metabolomics studies, high correlations with the intake of fish and seafood, and olive oil were expected, as they were mostly based on lipid metabolites [20, 50]. Future studies with a more comprehensive metabolomic analysis combining endogenous and exogenous metabolites are still warranted. We expect that the inclusion of more dietary biomarkers with higher specificity would improve the assessment of MD adherence and would reflect better its potential health benefits [51]. In our score, total polyphenols, selenium, linolenic acid, and carotenoids were grouped as dietary biomarkers of vegetables, fruits and nuts, legumes and cereals altogether because these dietary biomarkers are present at different concentrations across these highly-heterogenous food groups and one-to-one relationships can not be established. The analysis of interactions allowed us to detect that impaired renal function affected the association between the dietary biomarker-MDS and all-cause mortality, probably through its influence in the excretion of urinary dietary biomarkers. Further studies are needed to develop more robust adherence scores from dietary biomarker concentrations that may not be affected by impaired renal function.

The present findings on dietary biomarker-MDS are in accordance with previous InCHIANTI results showing that PUFA and total polyphenols inverse associations with overall mortality were only significant using dietary biomarkers but not using dietary questionnaires [13, 14]. Moreover, the metabolite score developed by Li et al. [20] was associated with CVD events independently of the MDS based on the FFQ. The explanation of why dietary biomarker-MDS was significantly associated with all-cause mortality, while the FFQ-MDS was not, might be related to the ability of dietary biomarkers to better address the complex diet-health relationship [51]. Furthermore, a dietary biomarker may better capture dietary exposure accounting for interindividual variations in different age-related changes.

The main strengths of this study is its longitudinal design, long follow-up, and the use of dietary biomarkers that reduce the potential dietary assessment errors of FFQ-based data. We also included repeated measures of the FFQ-MDS in the analysis as older adults are susceptible to change their dietary habits due to various conditions influenced by physiologic, pathologic, and/or psychologic factors [52]. In addition, we used a genetic score and a parental longevity score to better understand the predisposed mortality risks. Last, we used one of the common definitions of MDS [10], facilitating the comparison with results from other studies [53]. However, this investigation also has some limitations. Firstly, we only had baseline measurements of the dietary biomarkers, and their stability over time in this cohort is uncertain. However, in other longitudinal studies dietary biomarkers like plasma carotenoids, total SFA, MUFA and PUFA were reported to be stable, with an intraclass correlation coefficient ranging between 0.50–0.68 over 3 to 15 years apart [54–56]. Taking into consideration that FFQ-MDS slightly changed across follow-ups, we may assume similar changes for the dietary biomarker MDS. Secondly, there are more specific dietary biomarkers for some MD food groups as described in the literature [16, 18, 57–59], but they were not available in our cohort. In the present study, the panel of dietary biomarkers was selected based on a literature search and an a posteriori validation through correlation analyses. However, the existence of multiple food sources affecting the levels of these dietary biomarkers may have reduced the specificity of the present score for the Mediterranean diet. Indeed, the correlation coefficient and level of agreement between the FFQ- and dietary biomarker-MDS was low. Thirdly, although we adjusted our model by several potential confounders, residual confounding cannot be ruled out. Last, our results require confirmation in other populations from different geographical regions.

## Conclusions

Adherence to MD assessed by a dietary biomarker panel based on key MD food groups, but not using a traditional FFQ, was inversely associated with long-term mortality in older adults. The linear dose-response between the dietary biomarker-MDS and mortality further supports its use in long follow-up evaluations to monitor the potential health benefits associated with MD. Finally, we would like to highlight the use of dietary biomarkers to improve nutritional assessment and to guide individualized dietary counseling to older people.

## Supplementary Information

**Additional file 1 **— .doc file including. **Supplementary Table S1**. STROBE-nut checklist. **Supplementary Table S2**. Baseline data on dietary intake and dietary biomarkers by dietary biomarkers-MDS tertiles. Data shown as median (p25, p75). **Supplementary Table S3**. Baseline data on dietary intake and dietary biomarkers by FFQ-MDS tertiles. Data shown as median (p25, p75). **Supplementary Table S4**. Association between MDS and individual components of dietary biomarker-MDS (as tertiles), and all-cause mortality in the InCHIANTI Study. *Resveratrol was categorized in two groups: moderate vs. no or high consumers. EPA, eicosapentaenoic acid; DHA, docosahexaenoic acid; MUFA, monounsaturated fatty acids; SFA, saturated fatty acids. Total number of deaths, 435. Base model was adjusted for age and sex. The fully-adjusted model included sex, age, BMI, education, smoking status, physical activity, impaired renal function, diabetes mellitus, chronic obstructive pulmonary disease, hypertension, cardiovascular disease, cancer, dementia, Parkinson disease, and energy intake. **Supplementary Table S5**. Association between MDS and individual components of dietary biomarker-MDS (as tertiles), and CVD mortality in the InCHIANTI Study. *Resveratrol was categorized in two groups: moderate vs. no or high consumers. EPA, eicosapentaenoic acid; DHA, docosahexaenoic acid; MUFA, monounsaturated fatty acids; SFA, saturated fatty acids. Total number of cardiovascular deaths, 139. Base model was adjusted for age and sex. The fully-adjusted model included sex, age, BMI, education, smoking status, physical activity, impaired renal function, diabetes mellitus, chronic obstructive pulmonary disease, hypertension, cardiovascular disease, cancer, dementia, Parkinson disease, and energy intake. **Supplementary Table S6**. Association between MDS and individual components of dietary biomarkers-MDS (as tertiles), and cancer mortality in the InCHIANTI Study. *Resveratrol was categorized in two groups: moderate vs. no or high consumers. EPA, eicosapentaenoic acid; DHA, docosahexaenoic acid; MUFA, monounsaturated fatty acids; SFA, saturated fatty acids. Total number of Cancer deaths, 85. Base model was adjusted for age and sex. The fully-adjusted model included sex, age, BMI, education, smoking status, physical activity, impaired renal function, diabetes mellitus, chronic obstructive pulmonary disease, hypertension, cardiovascular disease, cancer, dementia, Parkinson disease, and energy intake. **Supplementary Fig S1**. Dose-response relationship between Mediterranean Diet Score (MDS) and all-cause mortality. Panel A, FFQ-MDS; Panel B, dietary biomarker-MDS. Cox regression models included sex, age, BMI, education, smoking status, physical activity, impaired renal function, diabetes mellitus, chronic obstructive pulmonary disease, hypertension, cardiovascular disease, cancer, dementia, Parkinson disease, and energy intake.

## Data Availability

The datasets used and/or analyzed during the current study are available from the responsible for the InCHIANTI study (Dr. Luigi Ferrucci) on reasonable request. Data of the InCHIANTI study is available to all researchers upon justified request using the proposal form available on the InChianti website (http://inchiantistudy.net/wp/how-to-submit-a-proposal/).

## Abbreviations

BMI: Body mass index
CI: Confidence interval
COPD: Chronic obstructive pulmonary disease
CVD: Cardiovascular disease
MDS: Dietary biomarker-mediterranean diet score
DHA: Docosahexaenoic acid
EPA: Eicosapentanoic acid
EPIC: European prospective study into cancer and nutrition
FFQ: Food frequency questionnaires
FFQ-MDS: Food frequency questionnaire-mediterranean diet score
HALE: Healthy aging: a longitudinal study in Europe
HR: Hazard ratio
HT: Hypertension
ICC: Intraclass correlation coefficient
ICD: International classification of diseases
InCHIANTI: *Invecchiare nel Chianti*
IRF: Impaired renal function
MD: Mediterranean diet
MDS: Mediterranean diet score
MUFA: Monounsaturated fatty acid
PUFA: Polyunsaturated fatty acid
SFA: Saturated fatty acid
STROBE-NUT: Strengthening the reporting of observational studies in epidemiology-nutritional epidemiology
