# Supplementary material for: Adherence to the Mediterranean diet assessed by a novel dietary biomarker score and mortality in older adults: the InCHIANTI cohort study
Source: BMC Med. 2021 Nov 24;19:280. doi: 10.1186/s12916-021-02154-7 (PMC8611910; doi:10.1186/s12916-021-02154-7)
Supplement: Supplementary file 1 — Additional file 1 — .doc file including. Supplementary Table S1. STROBE-nut checklist. Supplementary Table S2. Baseline data on dietary intake and dietary biomarkers by dietary biomarkers-MDS tertiles. Data shown as median (p25, p75). Supplementary Table S3. Baseline data on dietary intake and dietary biomarkers by FFQ-MDS tertiles. Data shown as median (p25, p75). Supplementary Table S4. Association between MDS and individual components of dietary biomarker-MDS (as tertiles), and all-cause mortality in the InCHIANTI Study. *Resveratrol was categorized in two groups: moderate vs. no or high consumers. EPA, eicosapentaenoic acid; DHA, docosahexaenoic acid; MUFA, monounsaturated fatty acids; SFA, saturated fatty acids. Total number of deaths, 435. Base model was adjusted for age and sex. The fully-adjusted model included sex, age, BMI, education, smoking status, physical activity, impaired renal function, diabetes mellitus, chronic obstructive pulmonary disease, hypertension, cardiovascular disease, cancer, dementia, Parkinson disease, and energy intake. Supplementary Table S5. Association between MDS and individual components of dietary biomarker-MDS (as tertiles), and CVD mortality in the InCHIANTI Study. *Resveratrol was categorized in two groups: moderate vs. no or high consumers. EPA, eicosapentaenoic acid; DHA, docosahexaenoic acid; MUFA, monounsaturated fatty acids; SFA, saturated fatty acids. Total number of cardiovascular deaths, 139. Base model was adjusted for age and sex. The fully-adjusted model included sex, age, BMI, education, smoking status, physical activity, impaired renal function, diabetes mellitus, chronic obstructive pulmonary disease, hypertension, cardiovascular disease, cancer, dementia, Parkinson disease, and energy intake. Supplementary Table S6. Association between MDS and individual components of dietary biomarkers-MDS (as tertiles), and cancer mortality in the InCHIANTI Study. *Resveratrol was categorized in two groups: moderate vs. no o [file 12916_2021_2154_MOESM1_ESM.docx]

**Additional file 1**

**Adherence to Mediterranean diet assessed by a novel dietary biomarker panel and mortality in older adults: the InCHIANTI cohort study**

Nicole Hidalgo-Liberona, Tomás Meroño, Raúl Zamora-Ros, Montse Rabassa, Richard Semba, Toshiko Tanaka, Stefania Bandinelli, Luigi Ferrucci, Cristina Andrés-Lacueva, Antonio Cherubini

**S1 Table. STROBE-nut checklist.**

**S2 Table. Baseline data on dietary intake and dietary biomarkers by dietary biomarkers-MDS tertiles.** Data shown as median (p25, p75).

**S3 Table. Baseline data on dietary intake and dietary biomarkers by FFQ-MDS tertiles.** Data shown as median (p25, p75).

**S4 Table. Association between MDS and individual components of dietary biomarker-MDS (as tertiles), and all-cause mortality in the InCHIANTI Study.** *Resveratrol was categorized in two groups: moderate *vs.* no or high consumers. EPA, eicosapentaenoic acid; DHA, docosahexaenoic acid; MUFA, monounsaturated fatty acids; SFA, saturated fatty acids. Total number of deaths, 435. Base model was adjusted for age and sex. The fully-adjusted model included sex, age, BMI, education, smoking status, physical activity, impaired renal function, diabetes mellitus, chronic obstructive pulmonary disease, hypertension, cardiovascular disease, cancer, dementia, Parkinson disease and energy intake. Scores for SFA and Vitamin B12 were inverted from highest to lowest to compose the dietary biomarker-MDS.

**S5 Table. Association between MDS and individual components of dietary biomarker-MDS (as tertiles), and CVD mortality in the InCHIANTI Study.** *Resveratrol was categorized in two groups: moderate *vs.* no or high consumers. EPA, eicosapentaenoic acid; DHA, docosahexaenoic acid; MUFA, monounsaturated fatty acids; SFA, saturated fatty acids. Total number of cardiovascular deaths, 139. Base model was adjusted for age and sex. The fully-adjusted model included sex, age, BMI, education, smoking status, physical activity, impaired renal function, diabetes mellitus, chronic obstructive pulmonary disease, hypertension, cardiovascular disease, cancer, dementia, Parkinson disease and energy intake. Scores for SFA and Vitamin B12 were inverted from highest to lowest to compose the dietary biomarker-MDS.

**S6 Table. Association between MDS and individual components of dietary biomarkers-MDS (as tertiles), and cancer mortality in the InCHIANTI Study.** *Resveratrol was categorized in two groups: moderate *vs.* no or high consumers. EPA, eicosapentaenoic acid; DHA, docosahexaenoic acid; MUFA, monounsaturated fatty acids; SFA, saturated fatty acids. Total number of Cancer deaths, 85. Base model was adjusted for age and sex. The fully-adjusted model included sex, age, BMI, education, smoking status, physical activity, impaired renal function, diabetes mellitus, chronic obstructive pulmonary disease, hypertension, cardiovascular disease, cancer, dementia, Parkinson disease and energy intake. Scores for SFA and Vitamin B12 were inverted from highest to lowest to compose the dietary biomarker-MDS.

**S1 Fig. Dose-response relationship between Mediterranean Diet Score (MDS) and all-cause mortality. Panel A, FFQ-MDS; Panel B, dietary biomarkers-MDS.** Cox regression models included sex, age, BMI, education, smoking status, physical activity, impaired renal function, diabetes mellitus, chronic obstructive pulmonary disease, hypertension, cardiovascular disease, cancer, dementia, Parkinson disease and energy intake.

**S1 Table. STROBE-nut checklist.**

|  | Item No | Recommendation | STROBE-nut Recommendation | Reported on page # |
| --- | --- | --- | --- | --- |
| **Title and abstract** | 1 | (a) Indicate the study’s design with a commonly used term in the title or the abstract. | **nut-1.** State the dietary/nutritional assessment method(s) used in the title, abstract, or keywords. | Title p.1; Abstract p.3 |
|  |  | (b) Provide in the abstract an informative and balanced summary of what was done and what was found. |  | Abstract p.3 |
| Introduction | | |  |  |
| Background/rationale | 2 | Explain the scientific background and rationale for the investigation being reported. |  | Background p.5-6 |
| Objectives | 3 | State specific objectives, including any prespecified hypotheses |  | Background p.6 |
| Methods | | |  |  |
| Study design | 4 | Present key elements of study design early in the paper. |  | Methods p.6 |
| Setting | 5 | Describe the setting, locations, and relevant dates, including periods of recruitment, exposure, follow-up, and data collection. | **nut-5.** Describe any characteristics of the study settings that might affect the dietary intake or nutritional status of the participants, if applicable | Methods p.6 |
| Participants | 6 | (a) Cohort study—Give the eligibility criteria, and the sources and methods of selection of participants. Describe methods of follow-up  Case-control study—Give the eligibility criteria, and the sources and methods of case ascertainment and control selection. Give the rationale for the choice of cases and controls  Cross-sectional study—Give the eligibility criteria, and the sources and methods of selection of participants | **nut-6.** Report particular dietary, physiological, or nutritional characteristics that were considered when selecting the target population. | Methods p.6 |
|  |  | (b) Cohort study—For matched studies, give matching criteria and number of exposed and unexposed  Case-control study—For matched studies, give matching criteria and the number of controls per case |  |  |
| Variables | 7 | Clearly define all outcomes, exposures, predictors, potential confounders, and effect modifiers. Give diagnostic criteria, if applicable | **nut-7.1.** Clearly define foods, food groups, nutrients, or other food components.  **nut-7.2.** When using dietary patterns or indices, describe the methods to obtain them and their nutritional properties. | Methods p.7-9 |
| Data sources/ measurement | 8* | For each variable of interest, give sources of data and details of methods of assessment (measurement). Describe comparability of assessment methods if there is more than one group | **nut-8.1.** Describe the dietary assessment method(s), e.g., portion size estimation, number of days and items recorded, how it was developed and administered, and how quality was assured. Report if and how supplement intake was assessed.  **nut-8.2.** Describe and justify food composition data used. Explain the procedure to match food composition with consumption data. Describe the use of conversion factors, if applicable.  **nut-8.3.** Describe the nutrient requirements, recommendations, or dietary guidelines and the evaluation approach used to compare intake with the dietary reference values, if applicable.  **nut-8.4.** When using nutritional biomarkers, additionally use the STROBE Extension for Molecular Epidemiology (STROBE-ME). Report the type of biomarkers used and their usefulness as dietary exposure markers.  **nut-8.5.** Describe the assessment of nondietary data (e.g., nutritional status and influencing factors) and timing of the assessment of these variables in relation to dietary assessment.  **nut-8.6.** Report on the validity of the dietary or nutritional assessment methods and any internal or external validation used in the study, if applicable. | Methods (Frequency of consumption, Adherence to a MDS, dietary biomarkers, covariates) p.7-10 |
| Bias | 9 | Describe any efforts to address potential sources of bias | **nut-9.** Report how bias in dietary or nutritional assessment was addressed, e.g., misreporting, changes in habits as a result of being measured, or data imputation from other sources. | Use of dietary Biomarkers p.7-9 |
| Study size | 10 | Explain how the study size was arrived at |  | Methods p.6 |
| Quantitative variables | 11 | Explain how quantitative variables were handled in the analyses. If applicable, describe which groupings were chosen and why | **nut-11.** Explain the categorization of dietary/nutritional data (e.g., use of N-tiles and handling of nonconsumers) and the choice of reference category, if applicable. | Methods (Dietary markers assessment, statistical analysis) p. 7-11 |
| Statistical methods | 12 | (a) Describe all statistical methods, including those used to control for confounding | **nut-12.1.** Describe any statistical method used to combine dietary or nutritional data, if applicable.  **nut-12.2.** Describe and justify the method for energy adjustments, intake modeling, and use of weighting factors, if applicable.  **nut-12.3.** Report any adjustments for measurement error, i.e., from a validity or calibration study. | Methods (statistical analysis) p. 11-12 |
|  |  | (b) Describe any methods used to examine subgroups and interactions |  | Methods p.12 |
|  |  | (c) Explain how missing data were addressed |  | Methods p.6 |
|  |  | (d) Cohort study—If applicable, explain how loss to follow-up was addressed  Case-control study—If applicable, explain how matching of cases and controls was addressed  Cross-sectional study—If applicable, describe analytical methods taking account of sampling strategy |  |  |
|  |  | (e) Describe any sensitivity analyses |  | Methods p.12 |
| Results | | |  |  |
| Participants | 13* | (a) Report numbers of individuals at each stage of study—eg numbers potentially eligible, examined for eligibility, confirmed eligible, included in the study, completing follow-up, and analysed | **nut-13**. Report the number of individuals excluded based on missing, incomplete, or implausible dietary/nutritional data | Methods p.6  Results p.12 |
|  |  | (b) Give reasons for non-participation at each stage |  |  |
|  |  | (c) Consider use of a flow diagram |  | Fig 1. p 27 |
| Descriptive data | 14* | (a) Give characteristics of study participants (eg demographic, clinical, social) and information on exposures and potential confounders | **nut-14.** Give the distribution of participant characteristics across the exposure variables if applicable. Specify if food consumption of total population or consumers only were used to obtain results. | Results p.12-14  Table 2, p29 |
|  |  | (b) Indicate number of participants with missing data for each variable of interest |  |  |
|  |  | (c) Cohort study—Summarise follow-up time (eg, average and total amount) |  | Results p.14 |
| Outcome data | 15* | Cohort study—Report numbers of outcome events or summary measures over time |  | Results p.15-16 |
|  |  | Case-control study—Report numbers in each exposure category, or summary measures of exposure |  |  |
|  |  | Cross-sectional study—Report numbers of outcome events or summary measures |  |  |
| Main results | 16 | (a) Give unadjusted estimates and, if applicable, confounder-adjusted estimates and their precision (eg, 95% confidence interval). Make clear which confounders were adjusted for and why they were included | nut-16. Specify if nutrient intakes are reported with or  without inclusion of dietary supplement intake, if  applicable. | Results p.13-16 |
|  |  | (b) Report category boundaries when continuous variables were categorized |  | Results p.11  Table 2, p29 |
|  |  | (c) If relevant, consider translating estimates of relative risk into absolute risk for a meaningful time period |  |  |
| Other analyses | 17 | Report other analyses done—eg analyses of subgroups and interactions, and sensitivity analyses | nut-17. Report any sensitivity analysis (e.g., exclusion of  misreporters or outliers) and data imputation, if  applicable. | Results p.15-166 |
| Discussion | | |  |  |
| Key results | 18 | Summarise key results with reference to study objectives |  | Discussion p.16-18 |
| Limitations | 19 | Discuss limitations of the study, taking into account sources of potential bias or imprecision. Discuss both direction and magnitude of any potential bias | nut-19. Describe the main limitations of the data sources  and assessment methods used and implications for the  interpretation of the findings. | Discussion p.18-19 |
| Interpretation | 20 | Give a cautious overall interpretation of results considering objectives, limitations, multiplicity of analyses, results from similar studies, and other relevant evidence | nut-20. Report the nutritional relevance of the findings,  given the complexity of diet or nutrition as an exposure | Discussion p.18-19 |
| Generalisability | 21 | Discuss the generalisability (external validity) of the study results |  | Discussion p.19 |
| Other information | | |  |  |
| Funding | 22 | Give the source of funding and the role of the funders for the present study and, if applicable, for the original study on which the present article is based |  | Funding p.19-20 |
| Ethics |  |  | **nut-22.1. Describe the procedure for consent and study approval from ethics committee(s)** | Materials and methods  (Study design) p.4 |
| Supplementary Material |  |  | **nut-22.2.** Provide data collection tools and data as online material or explain how they can be accessed. | Availability of data and materials p. 19  Supplementary data p32-33 |

**S2 Table.** **Baseline data on dietary intake and dietary biomarkers by dietary biomarkers-MDS tertiles.**

|  | **All (n=642)** | **Tertile 1 (n=251)** | **Tertile 2 (n=193)** | **Tertile 3 (n=202)** | ***P*** |
| --- | --- | --- | --- | --- | --- |
|  |  |  |  |  |  |
| **FFQ-MDS** | **9 (7, 11)** | **8 (6, 10)** | **9 (8, 11)** | **10 (8, 11)** |  |
| Vegetables (g/d) | 166.9 (123.8, 243.1) | 157.3 (115.0, 230.5) | 170.3 (121.5, 246.7) | 184.0 (128.6, 260.1) | 0.013 |
| Legumes (g/d) | 15.3 (9.2, 22.4) | 13.6 (9.2, 22.4) | 15.5 (9.2, 25.7) | 15.8 (9.2, 22.4) | 0.45 |
| Fruit and nuts (g/d) | 277.8 (203.1, 367.6) | 259.4 (188.5, 341.6) | 288.3 (204.4, 364.6) | 287.1 (232.9, 400.4) | 0.001 |
| Cereals (g/d) | 202.8 (149.8, 260.3) | 190.5 (138.6, 251.7) | 205.8 (159.4, 262.8) | 210.8 (167.5, 275.6) | 0.008 |
| Fish and seafood (g/d) | 20.8 (12.0, 31.0) | 18.4 (10.7, 27.8) | 21.8 (13.2, 33.9) | 21.8 (14.3, 32.6) | 0.001 |
| Ratio of MUFAs/SFAs (g/d) | 1.5 (1.3, 1.7) | 1.5 (1.2, 1.7) | 1.5 (1.3, 1.7) | 1.5 (1.4, 1.8) | 0.004 |
| Meat and meat products (g/d) | 100.7 (76.3, 127.3) | 94.5 (74.8, 125.9) | 102.5 (79.1, 129.0) | 105.6 (76.1, 127.2) | 0.69 |
| Milk dairy products (g/d) | 168.1 (82.2, 221.1) | 158.0 (77.7, 218.3) | 169.0 (93.0, 222.1) | 172.0 (76.2, 220.6) | 0.83 |
| Alcohol (g/d) | 7.6 (0.0, 26.6) | 3.8 (0.0, 17.5) | 9.6(0. 2, 26.9) | 13.3 (1.3, 26.8) | <0.001 |
| **dBMK-MDS** | **9 (8, 11)** | **7 (6, 8)** | **9 (9, 10)** | **12 (11, 13)** |  |
| Total polyphenols (mg GAE/d) | 148.2 (119.8, 196,4) | 125.6 (99.7, 170.8) | 148.6 (112.5, 195.5) | 176.6 (132.8, 213.0) | <0.001 |
| Total carotenoids, (µmol/L) | 1.7 (1.4, 2.1) | 1.5 (1.2, 1.8) | 1.7 (1.4, 2.1) | 2.1 (1.7, 2.5) | <0.001 |
| Fatty acid C18:3 n, 3 (Linolenic Acid) (µmol/L) | 43.3 (31.9, 59.5) | 36.3 (29.3, 49.7) | 41.7 (30.5, 54.7) | 56.0 (40.6, 77.8) | <0.001 |
| EPA+DHA (µmol/L) | 140.1 (107.4, 177.1) | 114.9 (89.3, 147.1) | 138.5 (108.0, 170.3) | 168.3 (141.1, 197.7) | <0.001 |
| ratio of MUFAs/SFAs | 0.96 (0.87, 1.04) | 0.91 (0.84, 0.99) | 0.96 (0.89, 1.05) | 1.02 (0.92, 1.10) | <0.001 |
| Total SFAs (mmol/L) | 3.7 (3.2, 4.3) | 3.6 (3.1, 4.2) | 3.7 (3.1, 4.3) | 3.7 (3.3, 4.4) | 0.16 |
| Resveratrol phase II metabolites (nmol/24h) | 915.2 (0.0, 3255.0) | 155.7 (0.0, 2556.1) | 1462.6 (189.7, 4312.5) | 1444.8 (463.0, 3463.9) | <0.001 |
| Selenium (µg/L) | 74.9 (67.0, 82.6) | 71.6 (64.5, 77.2) | 75.0 (67.2, 82.8) | 80.2 (72.0, 86.8) | <0.001 |
| Vitamin B12 (pmol/L) | 279.0 (197.6, 381.5) | 327.1 (223.5, 444.0) | 267.9 (202.4, 355.2) | 247.5 (178.7, 327.3) | <0.001 |

Data presented as median (p25, p75)

**S3 Table. Baseline data on dietary intake and dietary biomarkers by** **FFQ-MDS tertiles.**

|  | **All (n=642)** | **Tertile 1 (n=191)** | **Tertile 2**  **(n=272)** | **Tertile 3**  **(n=191)** | ***P*** |
| --- | --- | --- | --- | --- | --- |
| **FFQ-MDS** | 9 (7, 11) | 6 (5, 7) | 9 (8, 10) | 12 (11, 13) |  |
| Vegetables (g/d) | 166.9 (123.8, 243.1) | 119.1 (90.6, 155.6) | 163.1 (130.7, 220.8) | 248.0 (194.8, 322.4) | <0.001 |
| Legumes (g/d) | 15.3 (9.2, 22.4) | 9.3 (6.2, 15.5) | 16.0 (9.3, 22.5) | 19.2 (13.3, 26.4) | <0.001 |
| Fruit and nuts (g/d) | 277.8 (203.1, 367.6) | 227.8 (164.6, 304.3) | 274.8 (208.8, 368.6) | 329.9 (263.7, 407.1) | <0.001 |
| Cereals (g/d) | 202.8 (149.8, 260.3) | 162.3 (127.0, 218.1) | 204.1 (157.3, 251.8) | 243.7 (186.3, 303.7) | <0.001 |
| Fish and seafood (g/d) | 20.8 (12.0, 31.0) | 14.7 (7.7, 22.7) | 21.1 (13.5, 32.6) | 25.8 (17.3, 36.2) | <0.001 |
| Ratio of MUFAs/SFAs (g/d) | 1.5 (1.3, 1.7) | 1.3 (1.1, 1.5) | 1.5 (1.3, 1.7) | 1.7 (1.6, 2.0) | <0.001 |
| Meat and meat products (g/d) | 100.7 (76.3, 127.3) | 93.2 (73.9, 120.9) | 105.8 (80.2, 130.1) | 102.3 (75.1, 129.0) | 0.09 |
| Milk dairy products (g/d) | 168.1 (82.2, 221.1) | 193.3 (134.1, 255.4) | 168.6 (73.9, 222.2) | 132.4 (49.4, 193.5) | <0.001 |
| Alcohol (g/d) | 7.6 (0.0, 26.6) | 0.6 (0.0, 13.5) | 9.6 (0.2, 26.5) | 13.4 (4.9, 27.1) | <0.001 |
| **dBMK-MDS** | 9 (8, 11) | 8 (7, 10) | 9 (8, 11) | 10 (8, 12) |  |
| Total polyphenols (mg GAE/d) | 148.2 (109.9, 196.5) | 141.1 (104.5, 197.4) | 144.6 (109.8, 180.1) | 162.5 (121.6, 213.5) | 0.011 |
| Total carotenoids, (µmol/L) | 1.7 (1.4, 2.1) | 1.6 (1.3, 2.0) | 1.8 (1.4, 2.1) | 1.8 (1.5, 2.3) | 0.001 |
| Fatty acid C18:3 n, 3 (Linolenic Acid) (µmol/L) | 43.3 (31.9, 59.5) | 42.9 (31.2, 57.4) | 42.1 (31.3, 59.6) | 44.8 (33.3, 64.3) | 0.20 |
| EPA+DHA (µmol/L) | 140.1 (107.4, 177.1) | 134.4 (109.2, 170.0) | 140.1 (101.5, 172.5) | 146.0 (114.3, 181.3) | 0.22 |
| ratio of MUFAs/SFAs | 0.96 (0.87, 1.04) | 0.93 (0.86, 1.02) | 0.96 (0.87, 1.03) | 0.97 (0.90, 1.09) | 0.001 |
| Total SFAs (mmol/L) | 3.7 (3.2, 4.3) | 3.7 (3.3, 4.4) | 3.7 (3.1, 4.2) | 3.7 (3.2, 4.3) | 0.29 |
| Resveratrol phase II metabolites (nmol/24h) | 915.2 (0.0, 3255.0) | 324.8 (0.0, 2636.5) | 1046.2 (0.0, 3043.0) | 1781.8 (427.7, 4604.8) | <0.001 |
| Selenium (µg/L) | 74.9 (67.0, 82.6) | 74.6 (66.2, 82.4) | 75.0 (67.5, 82.5) | 74.9 (66.0, 83.1) | 0.85 |
| Vitamin B12 (pmol/L) | 279.0 (197.6, 381.5) | 321.9 (205.7, 425.5) | 275.3 (199.4, 358.5) | 265.7 (188.7, 349.5) | 0.013 |

Data shown as median (p25, p75)

**S4 Table. Association between MDS and individual components of dietary biomarker-MDS (as tertiles), and all-cause mortality in the InCHIANTI Study.**

|  | **HR_T2_*_vs._*_T1_**  **(95%CI)** | **HR_T3_*_vs._*_T1_**  **(95%CI)** | **HR per unit increase†**  **(95%CI)** |
| --- | --- | --- | --- |
| **FFQ-MDS**  Base model  Final model | 1.15 (0.92, 1.44)  1.11 (0.89, 1.39) | 0.91 (0.70, 1.19)  0.90 (0.69, 1.19) | 1.01 (0.97,1.05)  1.01 (0.97, 1.05) |
| **dBMK-MDS**  Base model  Final model | 0.91 (0.73, 1.14)  0.91 (0.73, 1.15) | 0.66 (0.52, 0.83)  0.72 (0.56, 0.91) | 0.95 (0.92, 0.99)  0.96 (0.83, 0.99) |
| **Individual dBMK:** |  |  |  |
| **Total Polyphenols**  Base model  Final model | 0.78 (0.62,0.99)  0.82 (0.64, 1.03) | 0.80 (0.63,1.02)  0.77 (0.60, 0.98) | 0.71 (0.43, 1.16)  0.65 (0.39, 1.07) |
| **Carotenoids**  Base model  Final model | 0.91 (0.73, 1.15)  0.94 (0.75, 1.19) | 0.74 (0.59, 0.93)  0.80 (0.63, 1.02) | 0.86 (0.75, 1.00)  0.83 (0.81, 1.08) |
| **Linolenic acid**  Base model  Final model | 0.89 (0.71, 1.13)  0.87 (0.69, 1.10) | 0.82 (0.66, 1.03)  0.85 (0.67, 1.07) | 0.58 (0.38, 0.87)  0.62 (0.40, 0.95) |
| **Selenium**  Base model  Final model | 0.98 (0.78, 1.23)  0.90 (0.72, 1.13) | 0.75 (0.59, 0.95)  0.80 (0.62, 1.02) | 0.28 (0.09, 0.92)  0.31 (0.09, 1.11) |
| **EPA+DHA**  Base model  Final model | 0.66 (0.53, 0.84)  0.66 (0.52, 0.84) | 0.83 (0.67, 1.04)  0.83 (0.66, 1.05) | 0.57 (0.31, 1.02)  0.54 (0.30, 0.99) |
| **MUFA/SFA**  Base model  Final model | 1.03 (0.82, 1.30)  0.94 (0.74, 1.20) | 0.99 (0.79, 1.25)  0.91 (0.72, 1.16) | 0.89 (0.45, 1.78)  0.76 (0.37, 1.52) |
| **Resveratrol ^a^**  Base model  Final model | -  - | 1.05 (0.87, 1.26)  1.07 (0.88, 1.30) | 1.01 (0.93,1.10)  1.02 (0.93,1.11) |
| **SFA**  Base model  Final model | 0.79 (0.63, 1.00)  0.78 (0.61, 0.98) | 0.85 (0.68, 1.06)  0.80 (0.63, 1.01) | 0.55 (0.21,1.44)  0.42 (0.15,1.15) |
| **Vitamin B12**  Base model  Final model | 0.98 (0.78, 1.24)  1.07 (0.83, 1.37) | 1.11 (0.88, 1.41)  1.07 (0.84, 1.37) | 1.32 (0.91, 1.93)  1.02 (0.70, 1.49) |

*Resveratrol was categorized in two groups: moderate *vs.* no or high consumers. EPA, eicosapentaenoic acid; DHA, docosahexaenoic acid; MUFA, monounsaturated fatty acids; SFA, saturated fatty acids. Total number of deaths, 435. Base model was adjusted for age and sex. The fully-adjusted model included sex, age, BMI, education, smoking status, physical activity, impaired renal function, diabetes mellitus, chronic obstructive pulmonary disease, hypertension, cardiovascular disease, cancer, dementia, Parkinson disease and energy intake. Scores for SFA and Vitamin B12 were inverted from highest to lowest to compose the dietary biomarker-MDS. **†The following variables have been log-transformed for this analysis: Total polyphenols, linolenic acid, selenium, EPA+DHA, Resveratrol, SFA and Vitamin B12.**

**S5 Table. Association between MDS and individual components of dietary biomarker-MDS (as tertiles), and CVD mortality in the InCHIANTI Study.**

|  | **HR_T2_*_vs._*_T1_**  **(95%CI)** | **HR_T3_*_vs._*_T1_**  **(95%CI)** | **HR per unit† increase (95%CI)** |
| --- | --- | --- | --- |
| **FFQ-MDS**  Base model  Final model | 1.22 (0.83, 1.80)  1.20 (0.81, 1.78) | 0.99 (0.62, 1.62)  1.05 (0.64, 1.72) | 0.99 (0.93, 1.06)  0.99 (0.94, 1.07) |
| **dBMK-MDS**  Base model  Final model | 0.90 (0.61, 1.32)  0.88 (0.59, 1.30) | 0.55 (0.36, 0.85)  0.60 (0.38, 0.93) | 0.92 (0.86, 0.98)  0.93 (0.87, 0.99) |
| **Individual dBMK:** |  |  |  |
| **Total Polyphenols**  Base model  Final model | 1.05 (0.71, 1.56)  1.07 (0.71, 1.60) | 0.83 (0.52, 1.30)  0.79 (0.49, 1.26) | 0.67 (0.28, 1.58)  0.58 (0.24, 1.40) |
| **Carotenoids**  Base model  Final model | 0.80 (0.54, 1.18)  0.81 (0.54, 1.20) | 0.57 (0.37, 0.88)  0.60 (0.39, 0.93) | 0.80 (0.62, 1.04)  0.87 (0.67, 1.14) |
| **Linolenic acid**  Base model  Final model | 0.76 (0.51, 1.13)  0.75 (0.50, 1.13) | 0.65 (0.44, 0.98)  0.67 (0.44, 1.02) | 0.30 (0.15, 0.60)  0.31(0.15, 0.66) |
| **Selenium**  Base model  Final model | 1.21 (0.82, 1.77)  1.13 (0.76, 1.66) | 0.74 (0.47, 1.16)  0.74 (0.47, 1.18) | 0.13 (0.02, 0.91)  0.09 (0.01, 0.76) |
| **EPA+DHA**  Base model  Final model | 0.65 (0.43, 0.98)  0.69 (0.45, 1.05) | 0.76 (0.51, 1.14)  0.80 (0.53, 1.20) | 0.48 (0.17, 1.34)  0.52 (0.18, 1.51) |
| **MUFA/SFA**  Base model  Final model | 1.00 (0.66, 1.53)  0.98 (0.64, 1.51) | 0.99 (0.65, 1.50)  0.96 (0.63, 1.51) | 0.66 (0.19, 2.33)  0.58 (0.16, 2.07) |
| **Resveratrol ^a^**  Base model  Final model | -  - | 1.07 (0.76, 1.50)  1.00 (0.71, 1.42) | 1.01 (0.88, 1.19)  1.01 (0.86, 1.17) |
| **SFA**  Base model  Final model | 1.02 (0.68, 1.51)  1.06 (0.71, 1.59) | 0.81 (0.53, 1.23)  0.73 (0.47, 1.12) | 0.27 (0.05, 1.54)  0.17 (0.03, 0.96) |
| **Vitamin B12**  Base model  Final model | 0.94 (0.62, 1.42)  0.95 (0.62, 1.46) | 1.29 (0.86, 1.94)  1.11 (0.73, 1.70) | 2.20 (1.16, 4.16)  1.88 (0.99, 3.58) |

*Resveratrol was categorized in two groups: moderate *vs.* no or high consumers. EPA, eicosapentaenoic acid; DHA, docosahexaenoic acid; MUFA, monounsaturated fatty acids; SFA, saturated fatty acids. Scores for SFA and Vitamin B12 were inverted from highest to lowest. Total number of cardiovascular deaths, 139. Base model was adjusted for age and sex. The fully-adjusted model included sex, age, BMI, education, smoking status, physical activity, impaired renal function, diabetes mellitus, chronic obstructive pulmonary disease, hypertension, cardiovascular disease, cancer, dementia, Parkinson disease and energy intake. Scores for SFA and Vitamin B12 were inverted from highest to lowest to compose the dietary biomarker-MDS. **†The following variables have been log-transformed for this analysis: Total polyphenols, linolenic acid, selenium, EPA+DHA, Resveratrol, SFA and Vitamin B12.**

**S6 Table. Association between MDS and individual components of dietary biomarkers-MDS (as tertiles), and cancer mortality in the InCHIANTI Study.**

|  | **HR_T2_*_vs._*_T1_ (95%CI)** | **HR_T3_*_vs._*_T1_ (95%CI)** | **HR per unit increase†**  **(95%CI)** |
| --- | --- | --- | --- |
| **FFQ-MDS**  Base model  Final model | 1.11 (0.67, 1.85)  1.16 (0.69, 1.93) | 0.75 (0.41, 1.38)  0.82 (0.44, 1.52) | 1.00 (0.92, 1.08)  1.02 (0.94, 1.10) |
| **dBMK-MDS**  Base model  Final model | 1.01 (0.62, 1.66)  1.16 (0.70, 1.92) | 0.68 (0.39, 1.16)  0.77 (0.45, 1.34) | 0.97 (0.89, 1.05)  0.99 (0.91, 1.07) |
| **Individual dBMK:** |  |  |  |
| **Total Polyphenols**  Base model  Final model | 0.61 (0.36, 1.04)  0.64 (0.37, 1.10) | 0.67 (0.40, 1.13)  0.60 (0.35, 1.03) | 0.86 (0.27, 2.73)  0.71 (0.23, 2.24) |
| **Carotenoids**  Base model  Final model | 0.95 (0.58, 1.56)  1.06 (0.64, 1.75) | 0.63 (0.36, 1.09)  0.72 (0.41, 1.27) | 0.75 (0.53, 1.06)  0.84 (0.59,1.19) |
| **Linolenic acid**  Base model  Final model | 1.27 (0.74, 2.21)  1.21 (0.70, 2.11) | 1.38 (0.81, 2.35)  1.37 (0.80, 2.36) | 2.32 (0.96, 5.62)  2.40 (0.96, 5.99) |
| **Selenium**  Base model  Final model | 0.84 (0.50, 1.42)  0.79 (0.46, 1.34) | 0.89 (0.53, 1.50)  0.91 (0.54, 1.55) | 0.77 (0.04, 14.73)  1.09 (0.05, 23.35) |
| **EPA+DHA**  Base model  Final model | 0.92 (0.55, 1.55)  0.94 (0.55, 1.60) | 0.92 (0.54, 1.55)  0.94 (0.55, 1.59) | 0.66 (0.18, 2.39)  0.61 (0.16, 2.31) |
| **MUFA/SFA**  Base model  Final model | 0.88 (0.52, 1.47)  0.80 (0.47, 1.35) | 0.90 (0.53, 1.51)  0.87 (0.51, 1.49) | 1.46 (0.33, 6.53)  1.26 (0.28, 3.90) |
| **Resveratrol***  Base model  Final model | -  - | 1.28 (0.82, 2.06)  1.43 (0.91, 2.24) | 1.12 (0.92, 1.37)  1.16 (0.96, 1.42) |
| **SFA**  Base model  Final model | 1.24 (0.72, 2.14)  1.28 (0.73, 2.22) | 1.33 (0.78, 2.26)  1.24 (0.72, 2.15) | 2.54 (0.32, 20.30)  1.78 (0.21, 14.90) |
| **Vitamin B12**  Base model  Final model | 0.87 (0.52, 1.46)  0.81 (0.47, 1.37) | 0.95 (0.57, 1.61)  0.82 (0.48, 1.41) | 0.83 (0.36, 1.94)  0.66 (0.28, 1.57) |

*Resveratrol was categorized in two groups: moderate *vs.* no or high consumers. dBMK, dietary biomarkers; EPA, eicosapentaenoic acid; DHA, docosahexaenoic acid; MUFA, monounsaturated fatty acids; SFA, saturated fatty acids. Total number of Cancer deaths, 85. Base model was adjusted for age and sex. The fully-adjusted model included sex, age, BMI, education, smoking status, physical activity, impaired renal function, diabetes mellitus, chronic obstructive pulmonary disease, hypertension, cardiovascular disease, cancer, dementia, Parkinson disease and energy intake. Scores for SFA and Vitamin B12 were inverted from highest to lowest to compose the dietary biomarker-MDS. **†The following variables have been log-transformed for this analysis: Total polyphenols, linolenic acid, selenium, EPA+DHA, Resveratrol, SFA and Vitamin B12.**

**S1 Fig.**

**
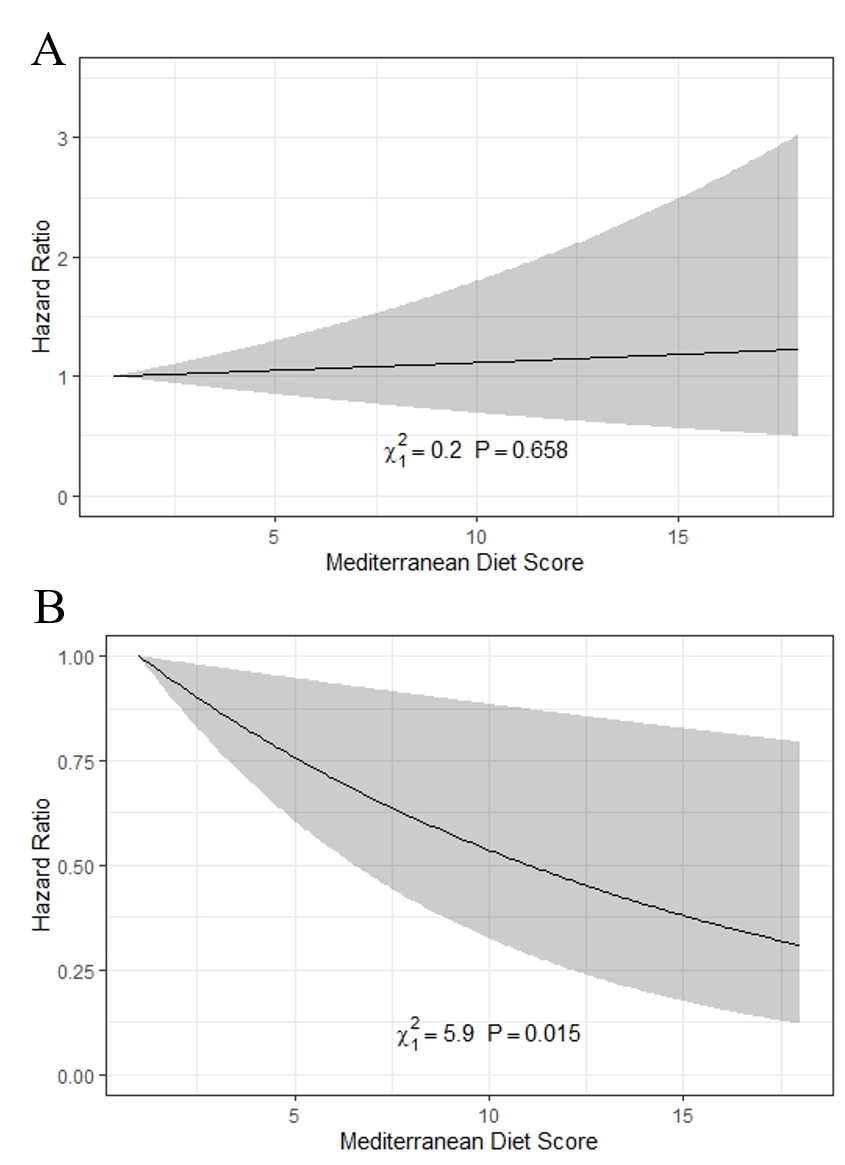
**

**S1 Fig.** Dose-response relationship between Mediterranean Diet Score (MDS) and all-cause mortality. Panel A, FFQ-MDS; Panel B, dietary biomarkers-MDS. Cox regression models included sex, age, BMI, education, smoking status, physical activity, impaired renal function, diabetes mellitus, chronic obstructive pulmonary disease, hypertension, cardiovascular disease, cancer, dementia, Parkinson disease and energy intake.
